# Profiling the mental health of diabetic patients: a cross-sectional survey of Zimbabwean patients

**DOI:** 10.1186/s13104-018-3881-9

**Published:** 2018-10-29

**Authors:** Alima M. Nyoni, Matthew Chiwaridzo, Catherine Tadyanemhandu, James January, Jermaine M. Dambi

**Affiliations:** 1 Department of Rehabilitation, College of Health Sciences, University of Zimbabwe, P.O Box A178, Avondale, Harare, Zimbabwe; 2 Department of Community Medicine, College of Health Sciences, University of Zimbabwe, P.O Box A178, Avondale, Harare, Zimbabwe; 3 School of Health and Rehabilitation Sciences, Faculty of Health Sciences, University of Cape Town Observatory, 7700 Cape Town, South Africa; 4 Department of Physiotherapy, School of Therapeutic Sciences, Faculty of Health Sciences, University of the Witwatersrand, Johannesburg, South Africa

**Keywords:** Diabetes, Mental health, Social support, Quality of life, Zimbabwe

## Abstract

**Objective:**

The burden of diabetes mellitus has exponentially increased in low resource settings. Patients with diabetes are more likely to exhibit poor mental health which negatively affects treatment outcomes. However, patients with high levels of social support (SS) are likely to report optimal mental health. We sought to determine how SS affects the report of psychiatric morbidity and health-related quality of life (HRQoL) in 108 diabetic patients in Harare, Zimbabwe.

**Results:**

The average age of participants was 54.1 (SD 18.6) years. Most of the participants were; females (69.4%), married (51.9%), and were of low level of income (43.5%). 37.1% of the participants exhibited signs of psychiatric morbidity [mean Shona Symptoms Questionnaire score—6.7 (SD 3.2)]. Further, patients also reported lower HRQoL [mean EQ-5D-VAS score—64.1 (SD 15.3)] and high levels of SS [mean Multidimensional Scale of Perceived Social Support score—43.7 (SD 11.5)]. Patients who received greater amount of SS had optimal mental health. Being female, unmarried, lower education attainment, having more comorbid conditions, being diagnosed with type 2 diabetes and having been diagnosed of diabetes for a longer duration were associated with poorer mental health. It is important to develop context-specific interventions to improve diabetic patients’ mental health.

**Electronic supplementary material:**

The online version of this article (10.1186/s13104-018-3881-9) contains supplementary material, which is available to authorized users.

## Introduction

The global burden of non-communicable diseases (NCDs) including diabetes has exponentially increased over the past few decades [1–7]. Accordingly, the worldwide prevalence of diabetes mellitus (DM) is projected to double by the year 2030 [1–3, 8]. Increasing globalisation, trends towards unhealthy diets, obesity, increased social inequality, and sedentary lifestyles have further exacerbated the worldwide burden of chronic NCDs [2, 4–10]. Unfortunately, the burden of diabetes is disproportionately substantial in low resource settings with 85% of all people with undiagnosed diabetes residing in low to middle-income countries [5–9]. For example, the burden of DM has significantly increased in Zimbabwe in the last 30 years, and its prevalence is currently conservatively pegged at 10% [11]. As in other low resource settings, a lack of resources and failure by governments to prioritise the screening and treatment of diabetes further perpetuates the pandemic [1–7, 12]. This is rather unfortunate as several systematic reviews and meta-analysis have shown that patients with diabetes are more likely to exhibit poor mental health [10–15]. For instance, the prevalence of depression is thrice in patients with diabetes as compared to the normative population [10, 13–15]. The burden of a chronic and life-long disorder predisposes patients to poor mental health functioning [16].

If unabated, negative mental health indices such as depression, anxiety and stress result in; poorer glycaemic control, decreased health-related quality of life (HRQoL), poorer adherence with DM treatment regime, treatment failure, increased odds of developing diabetes-related complications, increased health care utilisation and associated costs, and increased mortality [10, 15–20]. On the contrary, research suggests that an adequate amount of social support (SS) optimises treatment outcomes and patients’ mental health [10, 21]. For example, Tang et al. [22] carried out a cross-sectional survey of African-American diabetic patients (N = 89) to assess the relationship between SS and HRQoL. The study showed that SS; enhances self-management behaviours (healthy diet intake, frequent glucose level monitoring, consistent medication intake and regular physical activity engagement), and results in increased patient HRQoL. Social support can occur in various ways including; emotional, informational, financial, and affirmational support [22]. Although diabetes mellitus is one of the most prevalent NCDs [11], there is a lack of information on the mental health profile of patients from low-resource settings. This study, therefore, sought to assess the effects of social support on the levels of psychiatric morbidity and HRQoL of Zimbabwean diabetic patients.

## Main text

### Study settings

The study was carried at outpatient diabetic clinics at Parirenyatwa Group of Hospitals (PGH), Harare Central Hospital (HCH) and the Zimbabwe Diabetes Association (ZDA). PGH and HCH are the largest referral hospitals in Zimbabwe. The ZDA is a private facility which offers routine diabetic services including check-ups, medications supplies, health education and promotion talks, and community awareness and advocacy activities.

### Participants

Based on the study by Tang et al. [22] which yielded mean SS scores of 3.5 (SD = 1.3), we expected SS levels of Zimbabwean patients to be lower due to differences in socio-economic context, therefore assuming the following parameters; U_0_ = 3.5, U_1_ = 3.2, α = 0.05, β = 0.80, the minimum sample size was 150 participants. We consequently recruited adults diabetic patients (≥ 18 years) who were available during the study period and had given written consent. Diabetic patients with hearing impairments were excluded as we had no financial resources to hire interpreters. We also excluded patients who did not understand either English or Shona as study tools had been previously validated in Shona (a Zimbabwean native language) and English languages.

### Instruments

Participants’ characteristics, psychiatric morbidity, social support and HRQoL were measured using a purpose-built questionnaire, the Shona Symptom Questionnaire (SSQ), the Multidimensional Scale of Perceived Social Support (MSPSS), and the EQ-5D respectively. The demographic questionnaire extracted information on potential covariates, i.e. participants’ age, sex, educational level, marital status, financial status, type of diabetes, duration of diagnosis and comorbidities. Developed in Zimbabwe, the SSQ consists of local idioms and internationally recognised items expressive of common mental disorders (CMDs). The 14-item screen is especially sensitive in screening for depression and anxiety disorders [23]. The SSQ scoring is based on a binary response system, i.e. a yes response scores one point while a no response is equivalent to zero. A score of eight or higher on the SSQ indicates a high risk of psychiatric morbidity [24]. The MSPSS is a 12-item self-report measure of how one perceives their social support system, i.e. participants quantify support received from family, friends, and significant others. Items on the MSPSS are rated from one (strongly disagree) to five (strongly agree), the higher the score, the greater the social support [25]. The EQ-5D is a generic HRQoL which rates participants’ five dimensions, i.e. self-care, usual activities, pain/discomfort and anxiety/depression. Participants also rate their health on a visual analogue scale which ranges from zero (worst imaginable health state) to 100 (the best possible health state) [26, 27]. All study instruments have been previously validated in in the Zimbabwean context [24, 28, 29].

### Procedure

Approval to carry out the study was granted by; ZDA, HCH ethical committee and the Joint Research and Ethics Committee for the University of Zimbabwe, College of Health Sciences & Parirenyatwa Group of Hospitals (Ref: JREC/361/17). Prospective participants were approached as they awaited receiving services at the different clinical sites. The primary researcher (AMN) briefly explained the study aims and distributed the study pack which contained a detailed information letter, consent form and study outcome measures. Participants were required to give written consent before self-completing the study outcome measures. The researcher was available to attend to participants who had any queries and completed questionnaires were collected on the same day.

### Data analysis and management

Data were captured into Microsoft Excel and analysed using STATISTICA (version 14). Normality was checked using the Shapiro-Wilkin Test and; participants characteristics, EQ-5D, SSQ and MSPSS outcomes were summarised using descriptive statistics such as means and frequencies. Correlation co-efficiencies, Chi square/Fishers’ exact tests, and t-tests were used to determine the factors influencing patients’ mental health outcomes.

## Results

As seen in Table 1, the mean age of the participants was 54.1 (SD 18.6) years. Most participants; were female (69.4%), married (51.9%), educated (91.7%), employed (48.2%), and reported of below average income (53.7%), diagnosed with diabetes type 2 (55.6%), had been diagnosed for at least 6 years, and hypertension was the most common comorbid condition (46.3%). Significant other and friends were cited as the greatest and least sources of social support, and the mean social support (MSPSS) scores were 43.7 (SD 11.5). Participants’ mean HRQoL (EQ-5D utility & VAS) scores were 0.758 (SD 0.2) and 64.1 (SD 15.3) respectively. 37.1% of the participants exhibited signs of psychiatric morbidity, and the mean SSQ score was 6.7 (SD 3.2). Patients who received an adequate amount of social support reported lower psychiatric morbidity and greater HRQoL (see Table 2). Additionally, being female, unmarried, lower education attainment, having a greater number of comorbids, being diagnosed with type 2 diabetes and having been diagnosed of diabetes for a longer duration were associated with poorer mental health (see Table 3). Please refer to Additional files 1, 2, 3 for frequencies of responses on the MSPSS, EQ-5D and SSQ respectively.

**Table 1:** Descriptive statistics, N = 108

Variable	Attribute	Frequency n (%)
Gender	Female	75 (69.4)
	Male	33 (30.6)
Age^a^	Mean (SD)	54.1 (18.6)
Marital status	Single	16 (14.8)
	Married	56 (51.9)
	Widowed	36 (33.3)
Highest level of education	None	9 (8.3)
	Primary	20 (18.5)
	Secondary	47 (43.5)
	Tertiary	32 (29.6)
Employment status	Unemployed	35 (32.4)
	Formally employed	34 (31.5)
	Self-employed	18 (16.7)
	Retired	21 (19.4)
Perceived income	Very inadequate	11 (10.2)
	Inadequate	47 (43.5)
	Neutral	35 (32.4)
	Adequate	15 (13.9)
Diabetes type	Type 1	48 (44.4)
	Type 2	60 (55.6)
Years post diagnosis^a^	Median [Q_1_–Q_3_]	6 [3–15]
Comorbidities	Arthritis	10 (9.3)
	Hypertension	50 (46.3)
	HIV	5 (4.6)
	Ulcers	3 (2.8)
	Others	12 (11.1)
Social support (MSPSS) scores^a^	Family [mean (SD)]	4.0 (SD 1.1)
	Friends [mean (SD)]	2.8 (SD 1.3)
	Significant other [mean (SD)]	4.1 (SD 1.1)
	Summative score [mean (SD)]	43.7 (SD 11.5)
HRQoL (EQ-5D) scores^a^	Utility [mean (SD)]	0.758 (0.2)
	VAS score [mean (SD)]	64.1 (15.3)
Psychiatric morbidity (SSQ) scores^a^	SSQ scores ≥ 8 [n (%)]	40 (37.1)
	Summative score [mean (SD)]	6.7 (SD 3.2)

^a^Data not presented in the n (%) format

**Table 2:** Relationships between mental outcomes, N = 108

	MSPSS	SSQ	EQ-5D	EQ-5D
			Utility score	VAS score
MSPSS	1	Rho = − 0.190, p = 0.049	Rho = 0.240, p = 0.012	Rho = 0.242, p = 0.012
SSQ	Rho = − 0.190, p = 0.049	1	Rho = − 0.310, p < 0.001	Rho = − 0.380, p < 0.001
EQ-5D utility	Rho = 0.240, p = 0.012	Rho = − 0.310, p = 0.001	1	Rho = 0.422, p < 0.001
EQ-5D VAS	Rho = 0.242, p = 0.012	Rho = − 0.380, p < 0.001	Rho = 0.422, p < 0.001	1

**Table 3:** Determinants of mental health outcomes, N = 108

Variable	MSPSS	SSQ	EQ-5D	EQ-5D
			Utility score	VAS score
Age	Rho = − 0.1, p = 0.458	Rho = .09, p = 0.303	*Rho = − 0.4, p < 0.001	*Rho = − 0.3, p < 0.001
Gender	t (df = 106) = − 1.28, p = 0.203	*t (df = 106) = 3.9, p < 0.001	t (df = 106) = − 1.4 p = 0.203	*t (df = 106) = − 1.8, p = 0.078
Marital status	*X*^*2*^ (df = 3) = 6.2, p = 0.101	*X ^*2*^ *(df = 3) = 10.2, p = 0.0169*	*X ^2^ * (df = 3) = 28.7, p < 0.001*	*X*^*2*^ (df = 3) = 6.1, p = 0.11
Level of education	*X*^*2*^ (df = 3) = 3.9, p = 0.273	*X*^*2*^ (df = 3) = 2.6, p = 0.45	*X ^2^ * (df = 3) = 16.2, p = 0.001*	*X ^2^ * (df = 3) = 11.4, p = 0.01*
Employment status	*X ^*2*^ *(df = 4) = 9.8, p = 0.044*	*X*^*2*^ (df = 4) = 1.6, p = 0.807	*X ^2^ * (df = 4) = 22.7, p = 0.0001*	*X ^2^ * (df = 4) = 22.7, p = 0.0001*
Level of income	*X*^*2*^ (df = 4) = 1.1, p = .888	*X*^*2*^ (df = 4) = 4.8, p = .312	*X*^*2*^ (df = 4) = 8.2, p = 0.0847	*X ^2^ * (df = 4) = 9.8, p = 0.0444*
Diabetes duration	Rho = − 0.112, p = 0.250	Rho = 0.075, p = 0.442	*Rho = − 0.33, p < 0.001	*Rho = − 0.293, p = 0.002*
Type of diabetes	*t (df = 106) = 2.6, p = 0.009	t (df = 106) = 0.684, p = .496	*t (df = 106) = 2.9, p = 0.005	t (df = 106) = 1.4, p = 0.152
Comorbidities	Rho = − 0.11, p = 0.256	Rho = 0.210, p = 0.029	*Rho = − 0.447, p < 0.001	Rho = − 0.167, p = 0.083

* Flagged associations were statistically significant

## Discussion

The current study revealed that CMDs are prevalent in diabetic patients. Further, patients who received an adequate amount of social support had the best mental health outcomes, i.e. lower psychiatric morbidity and greater HRQoL, and this is congruent with previous studies [10, 21]. Most participants received their social support from their significant others, with the least support coming from friends. Many of the participants were married and therefore presumably received support from their spouses, and this may partially explain the discrepancies in sources of social support. However, there were subtle differences between support received from the family and significant other; which may be attributed to the Zimbabwean culture whereby it may be difficult for participants to succinctly distinguish between significant others and family as the two terms are often used interchangeably [29]. Further, patients who had more substantial financial resources reported of greater social support as having a stable source of income has been shown to be associated with the more considerable social network size [30].

When compared to the general population [31], patients with diabetes reported of lower HRQoL. The lower HRQoL can be attributed to pathological processes such as diabetic peripheral neuropathy (DPN). For example, DPN results in damage to peripheral nerves, which consequently leads to pain, reduced sensation and numbness, and these associated impairments negatively affect the patients’ HRQoL [32]. Further, restrictive treatment regimens (e.g. daily insulin shots and a strictly controlled diet free of refined and sugary foods) and DM-associated complications such as retinopathy and sexual dysfunction, may further negatively impact the HRQoL of DM patients and exacerbate their psychiatric morbidity [33–37]. More so, DM patients also reported depression, other studies have shown that diabetes and depression share the same pathophysiological/causal pathways [20]. Additionally, patients who had been diagnosed with DM for a more extended period also exhibited poorer HRQoL and greater psychiatric morbidity. The decreased mental health may be attributed to the increased burden imposed by complications of diabetes as the disease progresses over time [33, 37]. DM is, unfortunately, a progressive degenerative disease, i.e. the condition worsens with the passage of time regardless of treatment status [38]. Further, patients with type one diabetes reported of better HRQoL as compared to their type two counterparts. Type one diabetes usually affects the younger population who often have fewer disease-related complications [39]. Additionally, type one DM patients notably received more SS as compared to their type two counterparts. Type one diabetes has an earlier onset when the patient is still a minor, consequently, the immediate family is likely to be inclined/obliged to taking better care of minors and offer them more social support [40].

Studies suggest that a negative correlation exists between mental health issues and HRQoL of patients with diabetes as the complications and the burden that comes with DM exerts more psychological stress on individuals with diabetes thus increasing their risk of psychiatric morbidity [39, 41, 42]. Consistent with previous studies, the present study suggests that high levels of SS may result in the reduction of psychiatric morbidity and improved HRQoL in diabetic patients [10, 21, 22]. In the current study, female participants reported higher psychiatric morbidity. Previous studies have shown that compared to men, women are more likely to admit and open when something is wrong as compared to males who would prefer portraying themselves as healthy and “macho” [43]. This is, however, a priori, and further qualitative studies are warranted to understand the mental health of Zimbabwean patients with diabetes better. Additionally, married participants reported the least psychiatric morbidity scores; this is unsurprising given the buffering effect of SS on diabetes burden and diabetes-associated distress [44].

## Conclusion

The present study revealed that Zimbabwean DM patients reported poorer mental health and that patients who received an optimal amount of SS had the least psychiatric morbidity and greater HRQoL. Further, contextual factors, i.e. being female, unmarried, lower education attainment, having a higher number of comorbids, being diagnosed with type two (2) diabetes and having been diagnosed of diabetes for a longer duration were associated with poorer mental health. There is, therefore, need to routinely screen and appropriately refer patients with poor mental health for treatment. It is also essential to validate mental health outcomes in DM patients to increase mental health surveillance. Importantly, there is an enormous need to develop and implement context-specific interventions to improve the HRQoL of diabetic patients residing in low-resource settings.

## Limitations

The following methodological limitations are a threat to both the internal and external validity of the study outcomes:

- Data were collected cross-sectionally, causality cannot be inferred
- Participants were conveniently selected, there is therefore a possibility of selection bias
- Participants were only recruited from urban settings. However, 67% of the Zimbabwean population resides in rural areas [45]. Thus, outcomes may have limited generalisability
- We utilized generic mental health outcomes which may have limited content validity in unpacking mental health of DM patients
- Clinical data such as type and duration of diabetes was self-reported.

## Additional files

**Additional file 1.** Frequencies of responses on the MSPSS, N=108. Table denotes frequencies of responses on the MSPSS, a 12-item social support outcome measure. Responses are rated on a five-point Likert scale, ranging from strongly disagree=1 to strongly agree=5.

**Additional file 2.** Frequencies of responses on the EQ-5D, N=108. Table denotes frequencies of responses on the EQ-5D, a generic health-related quality of life measure. Respondents indicate whether they had problems in with self-care, usual activities, mobility, pain/discomfort and anxiety/depression on a three-adjunct scale. Responses are rated as “no problem”, “some problem” and “extreme problem”.

**Additional file 3.** Frequencies of responses on the SSQ, N=108. Table denotes frequencies of responses on the SSQ, a 14-item, binary common mental disorders (CMDs) screen. Respondents indicate if they had experienced any of the enlisted symptoms in the last seven days. A yes response is scored as “one” and no as “zero”, a score ≥ 8 is indicative of risk of CMD.

## Abbreviations

DM: diabetes mellitus
DPN: diabetic peripheral neuropathy
EQ-5D: European Quality of Life 5 Dimensions questionnaire
HCH: Harare Central Hospital
HRQoL: health-related quality of life
JREC: Joint Research and Ethics Committee for the University of Zimbabwe, College of Health Sciences & Parirenyatwa Group of Hospitals
MSPSS: Multidimensional Scale of Perceived Social Support
NCDs: non-communicable diseases
PGH: Parirenyatwa Group of Hospitals
SS: social support
SSQ: Shona Symptom Questionnaire
ZDA: Zimbabwe Diabetes Association
