# Supplementary material for: Profiling the mental health of diabetic patients: a cross-sectional survey of Zimbabwean patients
Source: BMC Res Notes. 2018 Oct 29;11:772. doi: 10.1186/s13104-018-3881-9 (PMC6206650; doi:10.1186/s13104-018-3881-9)
Supplement: Supplementary file 1 — Additional file 1. Frequencies of responses on the MSPSS, N=108. Table denotes frequencies of responses on the MSPSS, a 12-item social support outcome measure. Responses are rated on a five-point Likert scale, ranging from strongly disagree=1 to strongly agree=5. [file 13104_2018_3881_MOESM1_ESM.docx]

**Additional File 1: Frequencies of reported problems of the EQ-5D, N=108**

| Variable | Attribute | Frequency n, (%) |
| --- | --- | --- |
| Mobility | No problems | 68 (63.0) |
|  | Some problem | 38 (35.2) |
|  | Extreme | 2 (1.9) |
| Self-care | No problems | 92 (85.2) |
|  | Some problem | 16 (14.8) |
|  | Extreme | 0 (0) |
| Usual activities | No problems | 76 (70.4) |
|  | Some problems | 29 (26.9) |
|  | Extreme | 3 (2.8) |
| Pain/discomfort | No pain/discomfort | 32 (29.6) |
|  | Moderate | 67 (62.0) |
|  | Extreme | 9 (8.3) |
| Anxiety/depression | Not anxious/depressed | 37 (34.3) |
|  | Moderate | 67 (62.0) |
|  | Extreme | 4 (3.7) |
