# Supplementary material for: Profiling the mental health of diabetic patients: a cross-sectional survey of Zimbabwean patients
Source: BMC Res Notes. 2018 Oct 29;11:772. doi: 10.1186/s13104-018-3881-9 (PMC6206650; doi:10.1186/s13104-018-3881-9)
Supplement: Supplementary file 2 — Additional file 2. Frequencies of responses on the EQ-5D, N=108. Table denotes frequencies of responses on the EQ-5D, a generic health-related quality of life measure. Respondents indicate whether they had problems in with self-care, usual activities, mobility, pain/discomfort and anxiety/depression on a three-adjunct scale. Responses are rated as “no problem”, “some problem” and “extreme problem”. [file 13104_2018_3881_MOESM2_ESM.docx]

**Additional File 2: Frequencies of sources of social support on the MSPSS, N=108**

| Item | Strongly Disagree n (%) | Disagree n (%) | Neutral  n (%) | Agree  n (%) | Strongly Agree n (%) |
| --- | --- | --- | --- | --- | --- |
| 1.There is a special person who is around when I am in need | 1 (0.9) | 5 (4.6) | 12 (11.1) | 35 (32.4) | 52 (38.1) |
| 2. There is a special person with whom I can share joys and sorrows | 1 (0.9) | 2 (1.9) | 18 (16.7) | 31 (28.7) | 53 (49.1) |
| 3. My family really tries to help me | 1 (0.9) | 2 (1.9) | 12 (11.1) | 39 (36.1) | 51 (47.2) |
| 4. I get the emotional help & support I need from my family | 1 (0.9) | 3 (2.8) | 14 (13.0) | 41 (38.0) | 46 (42.6) |
| 5. I have a special person who is a real source of comfort to me | 3 (2.8) | 4 (3.7) | 16 (14.8) | 28 (25.9) | 54 (50.0) |
| 6. My friends really try to help me | 25 (23.1) | 11(10.2) | 28 (25.9) | 28 (25.9) | 13 (12.0) |
| 7. I can count on my friends when things go wrong | 26 (24.1) | 15(13.9) | 31 (28.7) | 25 (23.1) | 8 (7.4) |
| 8. I can talk about my problems with my family | 3 (2.8) | 6 (5.6) | 24 (22.2) | 30 (27.8) | 42 (38.9) |
| 9. I have friends with whom I can share my joys and sorrows | 23 (21.3) | 14(13.0) | 26 (24.1) | 22 (20.4) | 20 (18.5) |
| 10. There is a special person in my life who cares for my feelings. | 5 (4.6) | 1 (0.9) | 14 (13.0) | 30 (27.8) | 55 (50.9) |
| 11. My family is willing to help me make decisions | 3 (2.8) | 4 (3.7) | 24 (22.2) | 34 (31.5) | 40 (37.0) |
| 12. I can talk about my problems with my friends | 23 (21.3) | 16(14.8) | 32 (29.6) | 22 (20.4) | 12 (11.1) |
