# Supplementary material for: Profiling the mental health of diabetic patients: a cross-sectional survey of Zimbabwean patients
Source: BMC Res Notes. 2018 Oct 29;11:772. doi: 10.1186/s13104-018-3881-9 (PMC6206650; doi:10.1186/s13104-018-3881-9)
Supplement: Supplementary file 3 — Additional file 3. Frequencies of responses on the SSQ, N=108. Table denotes frequencies of responses on the SSQ, a 14-item, binary common mental disorders (CMDs) screen. Respondents indicate if they had experienced any of the enlisted symptoms in the last seven days. A yes response is scored as “one” and no as “zero”, a score ≥ 8 is indicative of risk of CMD. [file 13104_2018_3881_MOESM3_ESM.docx]

**Additional File 3: Frequencies of reported problems of the SSQ, N=108**

| Variable | No, n (%) | Yes, n (%) |
| --- | --- | --- |
| 1 Did you have times in which you were thinking deep or thinking about many things? | 28 (25.9) | 80 (74.1) |
| 2. Did you find yourself at sometimes failing to concentrate? | 50 (46.3) | 58 (53.7) |
| 3. Did you lose your temper of get annoyed over trivial matters? | 57 (52.8) | 51 (47.2) |
| 4. Did you have nightmare or bad dreams? | 70 (64.8) | 38 (35.2) |
| 5. Did you sometimes see or hear things which others would not see or hear? | 94 (87.0) | 14 (13.0) |
| 6. Was your stomach aching? | 64 (59.3) | 44 (40.7) |
| 7. Were you frightened by trivial things? | 61 (56.5) | 47 (43.5) |
| 8. Did you sometimes fail to sleep or lose sleep? | 16 (14.8) | 92 (85.2) |
| 9. Were there moments when you felt life was so tough that you cried or wanted to cry? | 43 (39.8) | 65 (60.2) |
| 10. Did you feel run down (tired)? | 23 (21.3) | 85 (78.7) |
| 11. Did you, at times, feel like committing suicide? | 98 (90.7) | 10 (9.3) |
| 12. Were you generally unhappy with things you were doing daily? | 59 (54.6) | 49 (45.4) |
| 13. Was your work lagging behind? | 56 (51.9) | 52 (48.1) |
| 14. Did you feel you had problems in deciding what to do | 65 (60.2) | 43 (39.8) |
